# Exogenous re-infection by a novel *Streptococcus pneumoniae *serotype 14 as a cause of recurrent meningitis in a child from The Gambia

**DOI:** 10.1186/1476-0711-8-3

**Published:** 2009-01-20

**Authors:** Martin Antonio, Claire Oluwalana, Ousman Secka, Tumani Corrah, Stephen Howie, Richard A Adegbola

**Affiliations:** 1 Bacterial Diseases Programme, Medical Research Council Laboratories, Banjul, The Gambia, Africa

## Abstract

We report a case of an infant who experienced exogenous re-infection of *Streptococcus pneumoniae *serotype 14 as a cause of recurrent meningitis after apparently successful antibiotic treatment with ceftriaxone. eBURST analysis revealed that isolates from the two episodes of meningitis belonged to hypervirulent ST63 and ST3321 clonal complexes respectively.

## Background

Pneumococcal meningitis is common within the African meningitis belt and occurs in a seasonal pattern indistinguishable from that of meningococcal meningitis [1]. *Streptococcus pneumoniae *can be subdivided by serological typing based on the capsular polysaccharide protein into at least 91 different serotypes [2] the majority of which rarely cause disease. *S. pneumoniae *serotype14 is a common cause of severe pneumococcal disease in The Gambia and ranks first among 127 pediatric invasive isolates recently tested during a 9-valent pneumococcal conjugate vaccine trial in The Gambia [3]. *S. pneumoniae *serotype14 has also been associated with meningitis outbreaks in Ghana [4] and Niger [5]. The World Health Organisation recommends the use of the 7-valent pneumococcal conjugate vaccine [6] which contains serotype 14, but this is not yet available for routine use in Africa including The Gambia. Multilocus sequence typing (MLST) is a well-established method that has been used to assess the population structure of *S. pneumoniae *during vaccine studies in The Gambia [3]. This report describes the use of MLST to demonstrate exogenous re-infection as a cause of recurrent meningitis in a child from The Gambia.

## Case report

A six months old female of the Manjago tribe, from a peri-urban coastal village in The Gambia was referred from a primary health care facility and admitted to the Royal Victoria Teaching Hospital, Banjul on 6^th ^December 2007 with clinical signs suggestive of meningitis. This diagnosis was confirmed by microscopic examination of the cerebrospinal fluid (CSF), which revealed gram-positive cocci and leucocytosis. The child was clinically successfully treated with intravenous ceftriaxone (100 mg/kg body weight daily for 13 days), during which *S. pneumoniae *was isolated from the CSF, subsequently identified as serotype 14 using methods described previously [7]. No neurological sequelae were evident after this episode. On 3^rd ^January 2008, the same child was re-admitted to the Medical Research Council Laboratories Hospital, Fajara again with clinical signs suggestive of meningitis. Similarly, this diagnosis was confirmed by microscopic examination of the CSF, which revealed gram-positive cocci and leucocytosis. Both CSF and blood culture from the second episode grew *S. pneumoniae *later confirmed as serotype 14. The second episode was treated without recurrence using intravenous chloramphenicol (25 mg/kg/dose) and crystalline penicillin (100,000 i.u/kg/dose) both 6 hourly for 14 days. Sadly the child had obvious neurological sequelae from the second episode.

The MRC microbiology laboratory submits to the external quality assurance programme of the United Kingdom National External Quality Assessment Service [8].

## Discussion

**Figure 1:**
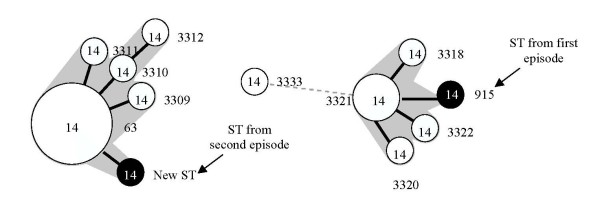
**Dendrogram showing the relatedness by BOX-PCR profile among the STs of *S. pneumoniae *serotype 14 used in this study**.

The fact that the serotyping results for *S. pneumoniae *were identical for the two episodes of meningitis was suggestive of endogenous (same isolate) reactivation due to failure of antibiotic treatment. The E-test MIC antibiotic susceptibility profile of the pre-treatment isolate from the first episode was sensitive to tetracycline and penicillin G whereas the two isolates from the pre-treatment second episode were both resistant to these antibiotics (table 1). Subsequent infection with more resistant organism may represent changes in the susceptibility of colonizing organism as a result of antibiotic therapy because carriage of *S. pneumoniae *serotype 14 is common in The Gambia [9]. Seemingly, *S. pneumoniae *carriage may provide a setting for selection of more resistant strains as well as providing continuing endogenous source of infection. To investigate this hypothesis, pneumococcal strain typing by BOX-PCR and MLST was performed as previously described [3] on the three isolates collected during the two episodes of meningitis. Sequence types (STs) were analyzed for relatedness using the eBURST v3 program [10]. MLST analysis showed that the pneumococcus isolated during the first episode of meningitis belonged to ST915 previously shown to be present in The Gambia [11]. However, MLST analysis from the pneumococci isolated from both blood and CSF during the second episode of meningitis were novel, i.e. not found in the *S. pneumoniae *MLST database [11]. An eBURST [10] analysis that included the STs from this study and its single variant locus (SVL) available in the MLST database [11] was performed using the stringent 6/7 identical loci definition. eBURST groups these STs into two major (ST63 and 3321) clonal complexes indicating that these two isolates are unrelated (figure 1).

**Table 1:** Antibiotic susceptibility patterns of *S. pneumoniae *serotype 14 used in this study.

		**E-Test MIC μg/ml**					
**Source**	**Episode**	**Penicillin G**	**Chloramphenicol**	**Tetracycline**	**Cotrimoxazole**	**Cefotaxime**	**Erythromycin**

Venous blood	Second	0.19	2	32	8	0.25	0.064

Cerebrospinal fluid	Second	0.19	1.5	24	6	0.25	0.125

Venous blood	First	0.016	2	0.19	6	0.032	0.125

**Figure 2:**
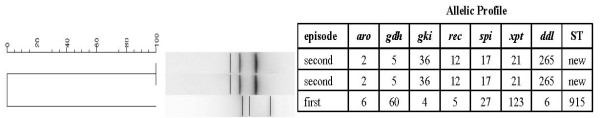
**Minimum spanning tree constructed using STs from the ST63 and ST3321 clonal complexes **[3]**including those identified in this study**. Each circle represents an ST. The area of each circle corresponds to the number of isolates. Thick, short, solid lines connect single-locus variants and thin, longer, solid lines connect double-locus variants. Unshaded (white) portions represent previously described Gambian STs and shaded (black) portion represents ST found in this study. Clonal complexes are shaded in grey

Serotype 14 is covered by both the 7-valent and 9-valent pneumococcal conjugate vaccines (Wyeth). However, during the efficacy trial in The Gambia, three of the children who received three doses of the 9-valent pneumococcal conjugate vaccine were infected with serotype 14 of ST 63, ST 3320 and ST 3333 [3]. In this study, the first episode of meningitis was caused by serotype 14 of ST915; a clone previously described in carriage from The Gambia and is a single-locus variant (SLV) to the ST3318 clonal complex which has so far only been reported from The Gambia [3]. The second episode was due to a novel ST (unassigned), which is a SLV to the hypervirulent ST63. The ST63 clonal complex has caused cases of meningitis in Northern Ghana and Niger [4,5]. However in The Gambia, ST63 is common in cases of pneumonia and bacteraemia [3]. Furthermore, ST63 can also express serotypes 15A, 19A, 19F and 23F [11] indicating it propensity to switch serotypes. The two clones (ST915 and unassigned ST) recovered from both episodes of meningitis shared no alleles and BOX-PCR fingerprinting analysis revealed distinct profiles indicating that these are unrelated clones (figures 1 and 2).

Despite *in vitro *resistance to tetracycline and penicillin G, both episodes of meningitis were successfully treated with regimens that include intravenous ceftriaxone (first episode) and crystalline penicillin and chloramphenicol (second episode). It is encouraging that in The Gambia, pneumococcal isolates are still sensitive to chloramphenicol, which remains a first line treatment for severe pneumonia, septicaemia and meningitis locally and is more affordable than cefotaxime or ciprofloxacin. Recurrent infections caused by organisms of the same species, sometimes with identical antibiotic susceptibility patterns are often regarded as relapse of persistent, unclear infection. Molecular genotyping techniques such as MLST have resulted in more accurate discrimination of strains thus allowing us to determine whether these recurrent infections are due to relapse or new infection. Our data demonstrate that the recurrent meningitis in this patient was due to new infection rather than to relapse. Importantly neither serological profile nor antibiotic susceptibility data were reliable predictors of strain difference or identity. Unrelated strains sometimes have the same or similar serological profiles while the same strains may have differences in either serological or antibiotic susceptibility. Typing of pneumococcal isolates by MLST is currently the only reliable means of distinguishing relapse from new infection, although other molecular-based methods may also be sufficiently discriminatory.

Recurrent systemic pneumococcal infection is known to occur in immunocompromised patients and patients with underlying conditions such as sickle-cell disease, asplenia, HIV, intracranial structural abnormalities or immununological abnormalities [12-15]. Unfortunately, we were not able to investigate any underlying conditions in this child.

## Conclusion

To our knowledge this is the first report describing the exogenous re-infection of serotype 14 pneumococcal disease as the cause of meningitis from The Gambia. The prominence of exogenous re-infection over relapse in this patient with meningitis shows that current therapy for meningitis in The Gambia is effective. However, this case re-emphasizes the need for prevention of invasive pneumococcal disease through routine conjugate pneumococcal vaccination, which has yet to be introduced anywhere in Africa. In addition, molecular genotyping of bacterial isolates is critical to understand recurrent infections in patients with meningitis.

## Consent

Consent was obtained from the patient's parents to report the case, which was identified through meningitis surveillance approved by The Gambian Government/MRC ethics committee

## Competing interests

The authors declare that they have no competing interests.

## Authors' contributions

MA, SH and RAA conceived the study. MA wrote the paper with contribution from CO, SH and RAA. OS and RA cultured and identified bacteria isolates from clinical samples. MA performed BOX-PCR and MLST molecular analysis. CO, SH and TC participated in clinical aspects of the study. All authors read and approved the final manuscript.

## References

[B1] Greenwood B (2006). 100 years of epidemic meningitis in West Africa – has anything changed?. Trop Med Int Health.

[B2] Park I, Pritchard D, Cartee R, Brandao A, Brandileone M, Nahm M (2007). Discovery of a new capsular serotype (6C) within serogroup 6 of Streptococcus pneumoniae. J Clin Microbiol.

[B3] Antonio M, Dada-Adegbola H, Biney E, Awine T, O'Callaghan J, Pfluger V, Enwere G, Okoko B, Oluwalana C, Vaughan A (2008). Molecular epidemiology of pneumococci obtained from Gambian children aged 2–29 months with invasive pneumococcal disease during a trial of a 9-valent pneumococcal conjugate vaccine. BMC Infect Dis.

[B4] Leimkugel J, Adams Forgor A, Gagneux S, Pfluger V, Flierl C, Awine E, Naegeli M, Dangy J, Smith T, Hodgson A (2005). An outbreak of serotype 1 Streptococcus pneumoniae meningitis in northern Ghana with features that are characteristic of Neisseria meningitidis meningitis epidemics. J Infect Dis.

[B5] Yaro S, Lourd M, Traore Y, Njanpop-Lafourcade B, Sawadogo A, Sangare L, Hien A, Ouedraogo M, Sanou O, Parent du Chatelet I (2006). Epidemiological and molecular characteristics of a highly lethal pneumococcal meningitis epidemic in Burkina Faso. Clin Infect Dis.

[B6] WHO (2007). Pneumococcal conjugate vaccine for childhood immunisation-WHO position paper. Wkly Epidemiol Rec.

[B7] Cutts F, Zaman S, Enwere G, Jaffar S, Levine O, Okoko J, Oluwalana C, Vaughan A, Obaro S, Leach A (2005). Efficacy of nine-valent pneumococcal conjugate vaccine against pneumonia and invasive pneumococcal disease in The Gambia: randomised, double-blind, placebo-controlled trial. Lancet.

[B8] United Kingdom National External Quality Assessment Service. http://www.ukneqas.org.uk.

[B9] Hill P, Akisanya A, Sankareh K, Cheung Y, Saaka M, Lahai G, Greenwood B, Adegbola R (2006). Nasopharyngeal Carriage of Streptococcus pneumoniae in Gambian Villagers. Clin Infect Dis.

[B10] eBURST V3. http://eburst.mlst.net.

[B11] Multi Locus Sequence Typing. http://spneumoniae.mlst.net/.

[B12] Mason EJ, Wald E, Tan T, Schutze G, Bradley J, Barson W, Givner L, Hoffman J, Kaplan S (2007). Recurrent systemic pneumococcal disease in children. Pediatr Infect Dis J.

[B13] Reinert R, Büssing A, Kierdorf H, Kühnemund O, Kaufhold A (1994). Recurrent systemic pneumococcal infection in an immunocompromised patient. Eur J Clin Microbiol Infect Dis.

[B14] Szabó J, Dobay O, Erdos M, Borbély A, Rozgonyi F, L M (2007). Recurrent infection with genetically identical pneumococcal isolates in a patient with interleukin-1 receptor-associated kinase-4 deficiency. J Med Microbiol.

[B15] Turvey S, Speert D (2007). Recurrent systemic pneumococcal disease and IRAK4 deficiency. Pediatr Infect Dis J.

